# Endoscopic Features of Gastric Mucosa-Associated Lymphoid Tissue Lymphoma without *Helicobacter pylori*

**DOI:** 10.3390/diagnostics14060607

**Published:** 2024-03-13

**Authors:** Mai Watanabe, Kouichi Nonaka, Maiko Kishino, Yoji Nagashima, Katsutoshi Tokushige

**Affiliations:** 1 Department of Digestive Endoscopy, Tokyo Women’s Medical University Hospital, 8-1 Kawada-Cho, Shinjuku-Ku, Tokyo 162-8666, Japan; watanabe.mai@twmu.ac.jp (M.W.); kishino.ige@twmu.ac.jp (M.K.); 2 Department of Surgical Pathology, Tokyo Women’s Medical University Hospital, 8-1 Kawada-Cho, Shinjuku-Ku, Tokyo 162-8666, Japan; nagashima.yoji@twmu.ac.jp; 3 Division of Gastroenterology, Tokyo Women’s Medical University Hospital, 8-1 Kawada-Cho, Shinjuku-Ku, Tokyo 162-8666, Japan; tokushige.ige@twmu.ac.jp

**Keywords:** gastric lymphoma, mucosa-associated lymphoid tissue, *Helicobacter pylori*, *HP*, MALT, pylori-negative, tree-like appearance, TLA, ballooning

## Abstract

Although gastric mucosa-associated lymphoid tissue (MALT) lymphoma without *Helicobacter pylori (HP)* has increased recently, a specific endoscopic classification has not been established; its endoscopic characteristics have not been investigated. In this study, we retrospectively investigated gastric MALT lymphoma without *HP* in our hospital and assessed differences in the endoscopic findings according to *HP* infection status. Fifty-seven patients with gastric MALT lymphoma Lugano stage I, diagnosed between January 2013 and March 2023, were divided into three groups (currently *HP* infected, previously infected, and uninfected), wherein their endoscopic findings were evaluated. Furthermore, the superficial type, as per the classification of Sano et al., was independently subdivided based on the endoscopic differential diagnoses, as follows: atrophic gastritis-like, angiodysplasia-like, superficial gastritis-like, and undifferentiated carcinoma-like. Compared with the currently infected group, the *HP*-uninfected group tended to have more small lesions without erosion and more discolored, undifferentiated carcinoma-like depressed lesions. In addition, the positive rate of the tree-like appearance (TLA) and ballooning characteristics of gastric MALT lymphoma in magnified findings was lower in the *HP*-uninfected group. In patients without *HP* infection, MALT lymphoma should be excluded, even in the absence of suspicious magnifying findings such as TLA or ballooning.

## 1. Introduction

Mucosa-associated lymphoid tissue (MALT) lymphoma was proposed by Isaacson et al. [1]. It is a low-grade lymphoma originating from B cells in the marginal region of lymphoid tissue in extra nodal organs such as the gastrointestinal tract, thyroid gland, and lungs, against a background of chronic inflammation [2,3].

Gastric malignant lymphoma often shows various endoscopic findings. The Sano classification [4] (superficial, ulcer, polypoid, fungated, and giant fold types) or that of Yao et al. [5] (superficial spreading, mass-forming, and giant fold types) are often used. However, there is no specific classification for the endoscopic images of gastric MALT lymphoma. Nakamura and Iida [6] classified the disease into four types, as follows: superficial (including gastritis-like, IIc-like depression, and multiple ulcer type), mass (including ulcer and submucosal tumor-like protuberance), diffuse infiltration, and mixed. Moreover, Oda et al. [7] classified the disease into redness/erosion, edema, IIc-like depression, ulceration, cobblestone appearance, protrusion, and white spot.

Approximately 80–90% of gastric MALT lymphomas are reportedly infected with *Helicobacter pylori* (*HP*) [8]. However, with the recent decline in *HP* infection rates, there has been an increase in the encountered cases of *HP*-negative gastric MALT lymphoma, leading to further diversification of their morphology. Therefore, this study aimed to determine the endoscopic features of gastric MALT lymphoma by comparing *HP*-uninfected cases with current *HP*-infection and previous *HP*-infection cases. This study advocated for original subdivisions that could be easily classified at endoscopy, discussing their characteristics. We suggest that the clarification of noteworthy endoscopic findings would greatly contribute to the diagnosis of MALT lymphoma in the future.

## 2. Materials and Methods

### 2.1. Study Design

Fifty-seven cases of gastric MALT lymphoma Lugano stage I [9], diagnosed at our hospital between January 2013 and March 2023, were included. A definitive diagnosis was established based on endoscopic biopsy, followed by histopathological examination, including immunostaining. Blood antibody tests and at least one of the following tests were performed to determine *HP* infection status: microscopy, urea breath, stool antigen, urinary antibody, and rapid urease tests. If two or more of these tests were positive, the patient was classified as currently infected. The patients were classified as uninfected if there was no atrophic gastritis on endoscopy and if two or more of the above *HP* infection tests, including the blood antibody test, were negative. Regarding the history of its eradication, cases of confirmed successful eradication plus one or more negative test results, including blood *HP* antibody test, were classified as previously infected. Even if two or more of the *HP* infection tests were negative, cases of clear endoscopic atrophic changes were classified as spontaneously eradicated cases in the previously infected group. At this time, we also tested for antibodies related to autoimmune gastritis and confirmed them to be negative. Furthermore, pathological examination confirmed the absence of parietal cell decrease. Cases of inadequate testing for *HP* infection status and cases of Lugano 2 or higher were excluded.

In each group, endoscopic findings, clinical course, and background information were retrieved from medical records, retrospectively reviewed, and compared. Informed consent for the study protocol was disclosed in the form of an opt-out page on the website available to all participants. The study was approved by the Ethics Committee of Tokyo Women’s Medical University Hospital (protocol code: 2022-0158).

### 2.2. Evaluation of Endoscopic Findings

The endoscopic images were examined at the time of diagnosis confirmation. The lesion site, number of lesions, presence or absence of erosions, and gross type were retrospectively assessed, and the gross type was classified according to the Sano classification [4]. These evaluations were performed by three Board Certified Gastroenterologists of The Japanese Society of Gastroenterology.

**Figure 1:**
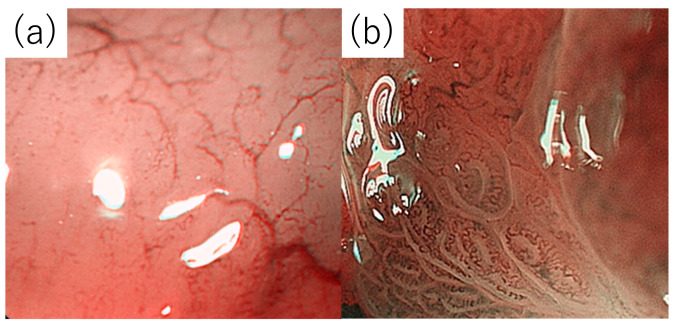
Magnified endoscopic findings of gastric mucosa-associated lymphoid tissue lymphoma. (**a**) Tree-like appearance: abnormal blood vessels branching like tree branches found in shiny mucous membranes without glandular structures. (**b**) Ballooning: swelling of surface ducts.

The magnified endoscopic findings were also evaluated using images combined with narrow-band imaging. The presence or absence of a tree-like appearance (TLA) and ballooning, which are considered characteristic endoscopic findings in patients with MALT lymphoma, was investigated (Figure 1).

### 2.3. Subdivision of Superficial Type Cases

Gastric MALT lymphoma has a relatively slow growth of tumor cells and many cases reportedly exhibit lesions corresponding to the superficial type according to the Sano [4] or Nakamura [6] classifications. The superficial type in this classification includes many endoscopic images. In this study, the superficial type was subdivided independently according to differential disease diagnoses, with reference to the existing classifications and their characteristics.

### 2.4. Statistical Analysis

All statistical data were analyzed using JMP Pro^®^ 16 (SAS Institute, Tokyo, Japan). Chi-square and Fisher’s exact tests were performed to compare categorical data and *t*-tests were employed for continuous data. In all cases, *p* < 0.05 was considered significant.

## 3. Results

### 3.1. Comparative Study of the Background and Endoscopic Findings

Of the 57 cases of MALT lymphoma diagnosed in this study, upper endoscopy was performed for the following indications: investigation of symptoms in 14 cases, screening in 33 cases, and other indications in 10 cases. The results of the currently infected, previously infected, and uninfected groups are presented in Table 1; overall, 28, 5, and 24 cases were classified in the currently infected, previously infected, and uninfected groups, respectively.

Table 1 presents the patients’ background characteristics and endoscopic image results by *HP* infection status.

The median ages of the currently infected, previously infected, and uninfected groups were 61, 60, and 62 years, respectively. There were no significant differences in the proportion of male patients among these groups.

Similarly, there were no significant differences in the site of the primary lesion among the three groups. Classifying the size of the primary lesion into large (≥30 mm), medium (10 ≤ *x* < 30 mm), and small (<10 mm), the uninfected group had the highest proportion of small lesions, at 41%, while the currently infected group had the highest proportion of large lesions, at 64% (*p* = 0.0039).

Regarding the number of lesions, 85.7% of the currently infected group comprised solitary cases. Similar to the currently infected group, there were more solitary cases rather than multiple cases in the uninfected group. However, the percentage remained at approximately 58.3%, lower than that in the currently infected group. In addition, there was a significant difference between the currently infected and uninfected groups in terms of erosions, with the currently infected group showing a higher incidence of erosions compared to the uninfected group, while no erosions were observed in the previously infected group (*p* = 0.0047).

When the primary lesions were classified according to the Sano classification, 82.4% were of the superficial type. Furthermore, when classified based on *HP* infection status, the superficial type was the most common in all groups and all cases in the previously infected group had this type. The ulcer and polypoid types accounted for 8.8% of all cases. The fungated and the giant fold types were observed in lesions ≥Lugano 2, and none were found in the Lugano 1 lesions, which were the subject of this study.

Regarding the magnified endoscopy findings, we examined the occurrence of TLA and ballooning, as shown in Figure 1. A TLA was observed in approximately 25 of the 40 cases (62.5%), wherein the presence could be determined endoscopically.

Table 2 details the results of the magnified endoscopic images by *H. pylori* infection status.

A TLA was observed in 93.8%, 40%, and 42% of the currently infected, previously infected, and uninfected groups, respectively. The TLA- and ballooning-positive rates were significantly lower in the uninfected group than in the currently infected group.

### 3.2. Comparative Study by Subdivision of Superficial Type Cases

**Figure 2:**
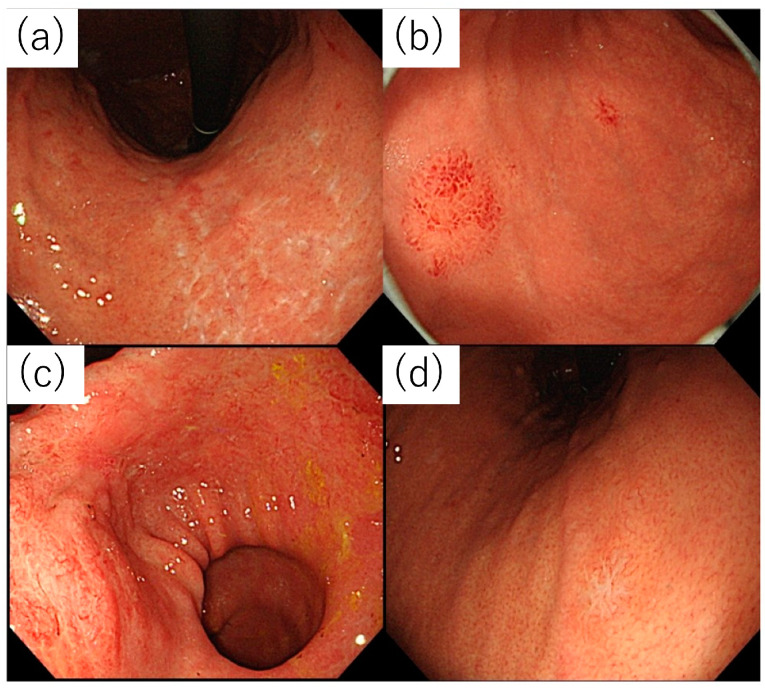
Subdivisions of the superficial type. (**a**) Atrophic gastritis-like. (**b**) Angiodysplasia-like. (**c**) Superficial gastritis-like. (**d**) Undifferentiated carcinoma-like.

There were 47 cases wherein the primary lesion was superficial, as follows: 21, 5, and 21 in the currently infected, previously infected, and uninfected groups, respectively. Atrophic gastritis-like lesions were defined as discolored, depressed lesions that were large, diffuse, and resembled atrophic gastritis, as shown in Figure 2a.

**Figure 3:**
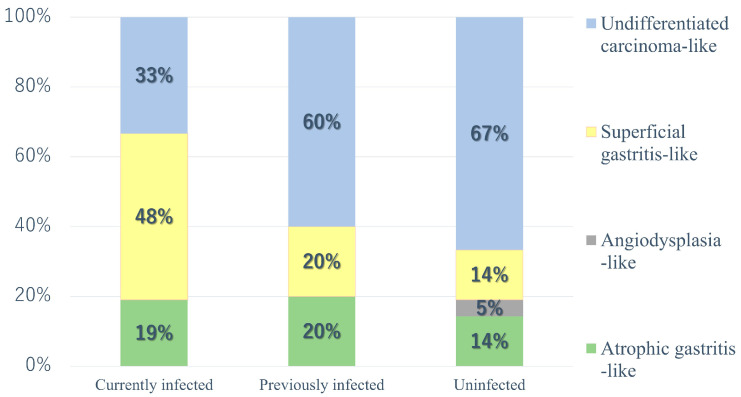
Relationship with *Helicobacter pylori (HP)* infection status in superficial type subdivisions. The *HP*-uninfected and previously infected groups were most likely to have undifferentiated carcinoma-like features, while the currently infected *HP* group was most likely to have superficial gastritis-like features.

In addition, as shown in Figure 2, lesions that were primarily reddish with conspicuous vasodilatation were classified as angiodysplasia-like (Figure 2b); those with mixed discoloration, redness, and uneven irregularities were defined as lesions that could be a differential diagnosis for superficial gastritis owing to inflammation, regardless of size, and were classified as superficial gastritis-like (Figure 2c); and discolored depressed small lesions suspected to be undifferentiated carcinoma were classified as undifferentiated carcinoma-like (Figure 2d). Finally, we decided that the case status was not significant. The results are summarized in Table 3 and are shown in Figure 3.

This table shows the proportions of groups subdivided into superficial types by *H. pylori* infection status.

As mentioned earlier, when we subdivided the surface layer type into four, the previously infected and uninfected groups had a higher proportion of undifferentiated carcinoma-like lesions than the currently infected group; angiodysplasia-like lesions were observed only in the uninfected group. In addition, the currently infected group had more superficial gastritis-like lesions than the uninfected group.

Furthermore, magnified endoscopic images were examined in each subgroup (Table 4). The TLA positivity rates were 87.5% and 42.1% in the superficial gastritis-like subtype (most prevalent in the currently infected group) and the undifferentiated carcinoma-like subtype (most prevalent in the uninfected group), respectively. The ballooning positivity rates were 71.4% and 21.1%, respectively.

**Figure 4:**
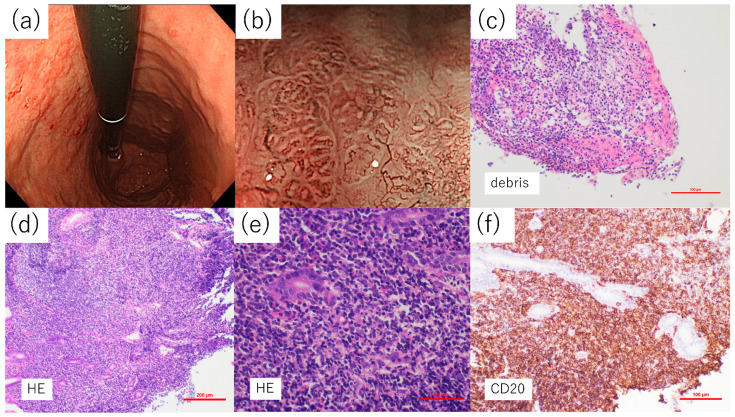
Example of the superficial gastritis-like subtype in those currently infected with *Helicobacter pylori.* (**a**) Rough and irregular mucosa with redness and discoloration is observed from the antrum to the lesser curvature of the upper body, along with multiple erosions. (**b**) Narrow band imaging-magnified observation revealed ductal dilatation and dendritic vessels in the lesion. (**c**) Aggregation of fibrin and inflammatory cells, which may be partly debris, is observed. (**d**) Hematoxylin-eosin staining shows lymphoma cells filling most of the specimen. (**e**) Lymphoepithelial lesion with lymphoma cells infiltrating the glands. (**f**) Most of the lymphocytes were CD20 positive.

Figure 4 shows examples of the superficial gastritis-like cases frequently observed in the *HP*-infected group.

The patient was a 74-year-old woman wherein an abnormality was detected during an upper endoscopy performed for a detailed examination of acid regurgitation symptoms. The background was an *HP*-infected stomach with diffuse redness and blood *HP* was antibody-positive. From the antrum to the lesser curvature of the upper body, a reddish and discolored irregular mucous membrane was observed, accompanied by multiple erosions. Magnifying endoscopy revealed shiny mucosa without glands, confirming a TLA. In addition, ballooning and duct dilatation were detected in the lesion. Histopathological findings indicated that CD20-positive lymphocytes were present under the epithelium, accompanied by many neutrophils. Furthermore, fibrin and inflammatory cell infiltration with debris were observed. Here, an image of a lymphoepithelial lesion (LEL) where lymphoma cells invaded the gland was observed (Figure 4e) and a MALT lymphoma diagnosis was made.

**Figure 5:**
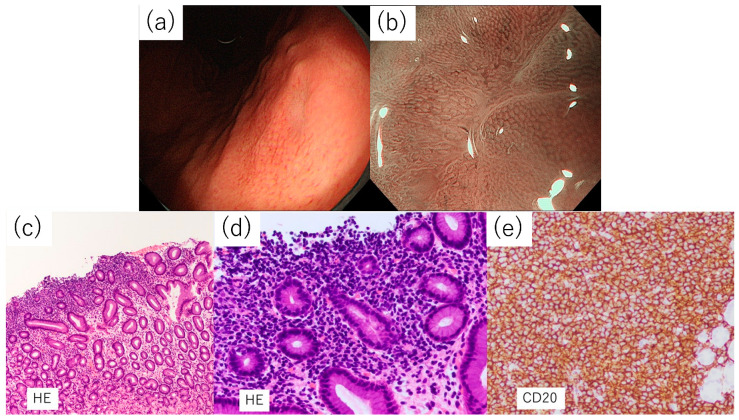
Example of the undifferentiated carcinoma-like subtype in a *Helicobacter pylori* uninfected patient. (**a**) A discolored, depressed lesion of approximately 5 mm was observed on the lesser curvature of the lower body of the stomach. Undifferentiated carcinoma was a differential diagnosis. (**b**) Narrow band imaging-magnified observation could not detect the tree-like appearance in the discolored depression. (**c**) Hematoxylin-eosin (HE) staining shows proliferation of lymphocytes just below the mucosa, but it is limited to the superficial layer of the lamina propria. (**d**) Proliferating lymphoma cells can be seen infiltrating the glands. (**e**) Many lymphocytes were CD20 positive.

Figure 5 shows the features of a case of a 58-year-old man with abnormalities detected during an upper endoscopy during a health checkup.

The background was an uninfected stomach without atrophy and an approximately 5 mm depressed lesion with a discolored tone was observed on the lesser curvature of the lower body, which required differentiation from undifferentiated carcinoma. The demarcation was ill-defined and there was no evidence of encroachment; therefore, MALT lymphoma was suspected rather than undifferentiated carcinoma, although it was a solitary lesion. Magnifying endoscopy findings did not reveal any abnormal blood vessels or ballooning suggestive of a TLA in the bleached area of the lesion. Furthermore, according to the pathological examination findings, unlike the case in Figure 4, where most of the specimen was filled with lymphocytes, lymphoma cells were partially aggregated and almost no inflammatory cells, such as neutrophils, were observed in the lesions. Most of the lymphocytes were CD20-positive B cells, and LEL was found in some, leading to the diagnosis of MALT lymphoma.

## 4. Discussion

In this study, the uninfected group tended to have more lesions without erosion and fewer lesions ≥30 mm than the currently infected group have. In addition, although solitary cases were the most frequent, the proportion of cases of multiple lesions was higher than that in the currently infected group. The superficial type was the most common in every group; the uninfected group had many discolored and depressed lesions requiring differentiation from undifferentiated carcinoma. One of the characteristics was the many cases of no characteristic magnifying endoscopy findings, such as TLA and ballooning.

According to the literature in Japan, the percentage of *HP*-negative MALT has recently been reported to be 10.5–54.3% [10] and is gradually increasing [11]. In our study, the combined ratio of the previously infected and uninfected groups accounted for approximately half of the total cases. It would be beneficial to clarify the endoscopic features of the *HP*-uninfected group, which may increase in the future and account for most gastric MALT lymphomas.

In addition, in previous endoscopic reports of *HP*-negative MALT lymphoma, the total number of cases of gastric MALT lymphoma at a single institution ranged between 17 and 158 [11,12,13,14,15]. The 57 patients in the present study represent a relatively large number of cases in a single center.

In addition, many previous reports treated the previously infected group of *HP*-negative MALT lymphoma similarly to the *HP*-uninfected group; there are few studies comparing the previously infected and uninfected groups. In this study, the previously infected group was treated as an independent group and compared with the uninfected group, and the treatment of the previously infected group was examined. The previously infected group showed a tendency similar to that of the uninfected group (e.g., many undifferentiated carcinoma-like lesions and few cases of erosion). However, the lesions in the previously infected group tended to concentrate in the middle and upper parts of the body—a finding different from those of the other groups. Owing to the limited sample size, accumulating more cases to determine whether the previously infected group could be treated as *HP*-negative is essential. The results may vary depending on the percentage of previously infected patients in the *HP*-negative group.

In a previous report, Tajika et al. [13] studied 120 cases of gastric MALT lymphoma and found that, in contrast to *HP*-positive cases with numerous eroded, ulcerative, and discolored regions, many *HP*-negative cases had cobblestone mucosa. Furthermore, they reported no differences between the two groups regarding lesion sites. Conversely, Nakamura et al. [14] reported that localization in the upper stomach and the macroscopic non-superficial type are common, infiltrates deeper than the submucosa, and LEL and lymphoid follicles are rare. In addition, Ono et al. [16] cited discolored areas and cobblestone-like mucosa as characteristics of *HP*-negative MALT lymphoma, consistent with the present study findings. However, Akamatsu et al. [17] reported no evident differences between *HP*-negative and *HP*-positive cases; no consensus has been reached.

Tajika et al. [13] considered that the characteristics of *HP*-negative MALT lymphoma differed from those of previous reports because the API2-MALT1 fusion gene was mixed in *HP*-negative MALT lymphoma. The API2-MALT1 translocation-negative group was non-responsive to eradication and had exophytic features. They reported that the API2-MALT1 translocation-positive group was non-responsive to eradication and was characterized by cobblestone mucosa.

Non-Helicobacter pylori Helicobacter (NHPH) infections, such as those due to *Helicobacter suis* and *Helicobacter heilmannii*, were recently reported to cause *HP*-negative MALT lymphoma. For example, NHPH infection was found in 19.6% of *HP*-negative cases [18] and 11.9% of *HP*-positive cases [19]. In addition, Takigawa et al. [20] reported that NHPH-infected gastric MALT lymphoma presenting with granular mucosa was a nodular gastritis-like MALT lymphoma, with relatively high specificity. In this study, superficial gastritis-like symptoms corresponding to the granular mucosa were found in approximately 20% of the infected and uninfected groups. However, in the *HP*-uninfected group of this study, discolored, depressed lesions were most frequent. The infection rates of NHPH and genetic testing were not available in this study, and the possibility that these and other unknown causes may be involved in a complex way cannot be ruled out. Further accumulation of cases and investigation of the causes are warranted.

In this study, each macroscopic type showed erosions, making it challenging to group the cases as a single classification. Previous reports included various treatments for erosions and the lack of consistency (e.g., ulcers and erosions being classified as one category or ulcers being classified separately) was a reason for the inconsistent results. In this study, ulcerative lesions were no longer classified as superficial and were excluded from the subdivision. Moreover, the currently infected group had a higher proportion of lesions with erosions compared to that in the uninfected and previously infected groups in this study. This may be attributed to a stronger infiltration of inflammatory cells, as observed in the cases depicted in Figure 4. Furthermore, the tendency for lesions with redness to be more prevalent in the currently infected group may also be attributed to similar reasons. On the contrary, differences in the positivity rates of a TLA and ballooning are noted as factors that inflammatory cell infiltration alone cannot fully explain.

Characteristic magnifying endoscopy findings of gastric MALT lymphoma include TLA and ballooning. Nonaka et al. [21] reported TLA as “abnormal branching blood vessels found in lustrous mucous membranes with loss of glandular structures,” a crucial finding in magnifying endoscopy for gastric MALT lymphoma. The authors reported that, in shiny mucosa without glandular structures, swelling of the intervening part between crypts was observed, depending on the degree of infiltration of the lamina propria by lymphoma cells. An intense infiltration causes the gland structure to disappear completely, restricting its confirmation. In addition, the appearance of abnormal, tree-like blood vessels is recognized at that site and, as the abnormal blood vessels and the glandular structure of the background mucosa tend to disappear, it aids in the differentiation from gastritis and venules in which branching blood vessels are observed [22].

Ono et al. [23,24,25] identified three features, as follows: (1) nonstructural area, (2) ballooning, and (3) abnormal vessels. Similar to a TLA, this ballooning is thought to be based on the degree of infiltration of the lamina propria by lymphoma cells.

In this study, the positivity rates of TLA and ballooning were significantly lower in the uninfected group than in the currently infected group. The undifferentiated carcinoma-like lesions, which were more common in the *HP*-uninfected group, tended to be less positive for TLA and ballooning. In addition to the fact that many undifferentiated carcinoma-like lesions are small, since the amount of lymphoma cell infiltration into the lamina propria is small, as shown in Figure 5c–e, detecting TLA and ballooning may be challenging. However, it may depend on the biopsy site of the lesion. In comparison, in the superficial gastritis-like lesion shown in Figure 4, lymphoma cells fill the lamina propria and both TLA and ballooning are observed in magnified endoscopic images. Moreover, Nonaka et al. reported the presence of abnormal micro vessels just beneath the mucosal epithelium in specimens from MALT lymphoma cases that underwent endoscopic mucosal resection [26]. We inferred this as abnormal blood vessels identical to a tree branch on NBI magnified findings. Nakamura et al. [27,28] reported that vascular endothelial growth factor (VEGF) activity was observed in gastric MALT lymphomas, leading to angiogenesis and the proliferation of microvascular networks, resulting in the development of a thick vascular system compared with healthy areas. VEGF activity in the fibroblasts surrounding tumor cells is reportedly associated with lymphoma growth [29]. Thus, confirming abnormal blood vessels may be challenging in lesions where lymphoma invasion is weak and relatively small.

A TLA is reportedly observed in 75% of gastric MALT lymphomas [30]. In this study, it was observed in 63% of cases. As mentioned above, since a TLA and ballooning were significantly observed in the currently infected group, it is suggested that the TLA-positivity rate may decrease as the uninfected group increases in the future.

In this study, undifferentiated carcinoma-like lesions were most prevalent in the *HP*-uninfected group. When discolored, depressed lesions are observed, it becomes important to differentiate between undifferentiated carcinoma and undifferentiated carcinoma-like gastric MALT lymphoma. Past reports have characterized undifferentiated carcinoma by solitary lesions, clear demarcation, evidence of encroachment, and distinctive vascular patterns such as wavy micro-vessels or a corkscrew pattern. On the contrary, MALT lymphoma is characterized by ill-demarcation, sometimes multiple lesions, and abnormal vessels such as those having a TLA [31]. These features aid in the differentiation between the two. However, distinguishing between them is not always straightforward. Particularly in this study, a TLA was observed in only approximately 42% of undifferentiated carcinoma-like lesions and the absence of characteristic expansive endoscopic findings does not negate the presence of MALT lymphoma. Therefore, a comprehensive consideration of other findings is necessary for a definitive diagnosis.

This study has some limitations. The first limitation is the bias in the assessment of endoscopic findings by the assessors. Gastric MALT lymphoma presents with a very diverse endoscopic picture, which makes classifying some cases difficult. In this study, endoscopic images were assessed and subdivided by three or more Board Certified Gastroenterologists of The Japanese Society of Gastroenterology to reduce this assessment bias. Another limitation is that the study was conducted at a single center. Although this study enrolled a relatively large number of cases for a single center study, it is still a small number overall. The target diseases were rare and further subdivision limited the number of cases in each group. Indeed, the very small number of angiodysplasia-like lesions made a comparison with other classifications difficult. A further study with more cases will confirm the findings of this study. Furthermore, *HP* infection status varies across regions and a further multicenter accumulation of cases is warranted in the future. In addition, in this study, the infection rate of NHPH and genetic testing was largely unperformed due to the limited availability of testing facilities domestically. However, as mentioned earlier, there is a possibility that these factors could be intricately involved as causes of *HP*-negative gastric MALT lymphoma. Further classification of these factors may lead to new discoveries regarding endoscopic features and treatment strategies.

## 5. Conclusions

Gastric MALT lymphoma without *HP* infection had more small lesions without erosion and undifferentiated carcinoma-like discolored, depressed lesions than the infected group. This result suggests that the positive rate of TLA and ballooning may be low. Regarding *HP* uninfected cases, differentiating gastric MALT lymphoma without magnified findings, such as a TLA and ballooning, is essential considering that endoscopic findings might show microscopic lesions. Since the number of cases of previously *HP*-infected gastric MALT lymphoma is small, a further accumulation of cases is needed to study the differences and features of the uninfected group and their endoscopic images.

## Figures and Tables

**Table 1:** Results by *H. pylori* infection status.

Characteristics No. (%)		Currently Infected Group (N = 28)	Previously Infected Group (N = 5)	Uninfected Group (N = 24)	Total (N = 57)
Sex	Male	16	3	10	29
		(57.1)	(60)	(41.7)	(50)
Median age (years)		61	60	62	61
Location in stomach	Upper body	7	1	4	12
		(25)	(20)	(16.7)	(21.1)
	Middle body	3	4	5	12
		(10.7)	(80)	(20.8)	(21.1)
	Lower body	11	0	7	18
		(39.3)	(0)	(29.2)	(31.6)
	Multiple locations	7	0	8	15
		(25)	(0)	(33.3)	(26.3)
Size	<10 mm	3	3	10	16
		(10.7)	(60)	(41.7)	(28.1)
	10–30 mm	7	0	9	16
		(25)	(0)	(37.5)	(28.1)
	≥30 mm	18	2	5	25
		(64.3)	(40)	(20.8)	(43.9)
No. of lesion	Solitary	24	2	14	40
		(85.7)	(40)	(58.3)	(70.2)
Erosion	Erosion present	13	0	2	15
		(46.4)	(0)	(8.3)	(26.3)
Macroscopic type	Superficial type	21	5	21	47
		(75)	(100)	(87.5)	(82.4)
	Ulcer type	4	0	1	5
		(14.2)	(0)	(4.2)	(8.8)
	Polypoid type	3	0	2	5
		(10.7)	(0)	(8.3)	(8.8)
	Fungated type	0	0	0	0
		(0)	(0)	(0)	(0)
	Giant fold type	0	0	0	0
		(0)	(0)	(0)	(0)

**Table 2:** Results of magnified endoscopic images by *H. pylori* infection status.

Endoscopic Features No. (%)		Currently Infected Group	Previously Infected Group	Uninfected Group	Total	Currently Infected Group vs. Uninfected Group *p*-Value
		N = 16	N = 5	N = 19	N = 40	
TLA	Yes	15	2	8	25	0.0016
		(93.8)	(40)	(42)	(62.5)	
		N = 15	N = 4	N = 18	N = 37	
Balloning	Yes	11	0	3	14	<0.001
		(73)		(16.7)	(38.9)	

TLA: tree-like appearance.

**Table 3:** Percentage of subdivision groups by *Helicobacter pylori* infection status.

Type No. (%)	Currently Infected Group N = 21	Previously Infected Group N = 5	Uninfected Group N = 21	Total N = 47	Currently Infected Group vs. Uninfected Group *p*-Value
Atrophic gastritis-like	4	1	3	8	n.s.
	(19)	(20)	(14)	(17)	
Angiodysplasia-like	0	0	1	1	n.s.
			(5)	(2.1)	
Superficial gastritis-like	10	1	3	14	0.0431
	(48)	(20)	(14)	(29.8)	
Undifferentiated carcinoma-like	7	3	14	24	0.0308
	(33)	(60)	(67)	(51.1)	

n.s.: not significant.

**Table 4:** Magnified endoscopic findings by superficial type subtypes.

Features No. (%)		Atrophic Gastritis-Like	Angiodysplasia-Like	Superficial Gastritis-Like	Undifferentiated Carcinoma-Like	Total
		N = 8	N = 1	N = 8	N = 19	N = 36
TLA	Yes	6	0	7	8	21
		(75)		(87.5)	(42.1)	(58.3)
		N = 6	N = 1	N = 7	N = 19	N = 33
Balloning	Yes	2	0	5	4	11
		(33.3)		(71.4)	(21.1)	(33.3)

## Data Availability

Data is contained within the article.
